# Brazilian Thoracic Society recommendations for the diagnosis and monitoring of asbestos-exposed individuals

**DOI:** 10.36416/1806-3756/e20240156

**Published:** 2024-07-29

**Authors:** Ubiratan Paula Santos, Eduardo Algranti, Eduardo Mello De Capitani, Gustavo Faibischew Prado, Ana Paula Scalia Carneiro, Sílvia Carla Sousa Rodrigues, Jefferson Benedito Pires de Freitas, Rodrigo Caruso Chate, Rafael Futoshi Mizutani, Hermano Albuquerque de Castro, Marcos Abdo Arbex, Patrícia Canto Ribeiro, Carlos Nunes Tietboehl, Maria Vera Cruz de Oliveira Castellano, Guilherme Ward Leite, Gustavo Corrêa de Almeida, Committee on Environmental and Occupational Diseases

**Affiliations:** 1. Grupo de Doenças Respiratórias Ocupacionais, Ambientais e de Cessação de Tabagismo, Divisão de Pneumologia, Instituto do Coracao - InCor - Hospital das Clinicas, Faculdade de Medicina, Universidade de Sao Paulo - FMUSP - Sao Paulo (SP) Brasil.; 2. Fundação Jorge Duprat Figueiredo de Segurança e Medicina do Trabalho - FUNDACENTRO - São Paulo (SP) Brasil.; 3. Disciplina de Pneumologia e Centro de informação e Assistência Toxicológica - CIATox - Departamento de Clínica Médica, Faculdade de Ciências Médicas, Universidade Estadual de Campinas - UNICAMP - Campinas (SP) Brasil.; 4. Hospital Alemão Oswaldo Cruz, São Paulo (SP) Brasil.; 5. Ambulatório de Pneumologia Ocupacional do SEST, Hospital das Clínicas, Universidade Federal de Minas Gerais - UFMG - Belo Horizonte (MG) Brasil.; 6. Serviço de Pneumologia, Hospital do Servidor Público Estadual Francisco Morato Oliveira (HSPE-FMO)/Instituto de Assistência Médica ao Servidor Público Estadual - IAMSPE - de São Paulo, São Paulo (SP) Brasil.; Instituto de Assistência Médica ao Servidor Público Estadual, São Paulo, SP, Brasil; 7. Departamento de Saúde Coletiva, Faculdade de Ciências Médicas da Santa Casa de São Paulo - FCMSCSP - São Paulo (SP) Brasil.; 8. Serviço de Radiologia, Instituto do Coracao - InCor - Hospital das Clinicas, Faculdade de Medicina, Universidade de Sao Paulo - FMUSP - Sao Paulo (SP) Brasil.; 9. Hospital Israelita Albert Einstein, São Paulo (SP) Brasil.; 10. Fundação Oswaldo Cruz - Fiocruz - Rio de Janeiro (RJ) Brasil.; 11. Área Temática Pneumologia, Faculdade de Medicina, Universidade de Araraquara, Araraquara (SP) Brasil.; 12. Atenção à saúde da Vice-Presidência de Ambiente Atenção e Promoção da Saúde, Fundação Oswaldo Cruz - Fiocruz - Rio de Janeiro (RJ) Brasil.; 13. Secretaria da Saúde do Estado do Rio Grande do Sul, Porto Alegre (RS) Brasil.; 14. Hospital de Base, Faculdade de Medicina de São José do Rio Preto, São José do Rio Preto (SP) Brasil.

**Keywords:** Asbestosis/diagnosis, Asbestosis/prevention & control, Environmental exposure, Occupational exposure

## Abstract

Asbestos was largely used in Brazil. It is a mineral that induces pleural and pulmonary fibrosis, and it is a potent carcinogen. Our objective was to develop recommendations for the performance of adequate imaging tests for screening asbestos-related diseases. We searched peer-reviewed publications, national and international technical documents, and specialists’ opinions on the theme. Based on that, the major recommendations are: Individuals exposed to asbestos at the workplace for ≥ 1 year or those with a history of environmental exposure for at least 5 years, all of those with a latency period > 20 years from the date of initial exposure, should initially undego HRCT of the chest for investigation. Individuals with pleural disease and/or asbestosis should be considered for regular lung cancer monitoring. Risk calculators should be adopted for lung cancer screening, with a risk estimate of 1.5%.

## INTRODUCTION

Asbestos is a general term that refers to a heterogeneous group of natural minerals that occur in the form of fibers (length to diameter ratio ≥3:1), mostly composed of hydrated magnesium silicates and a variable content of other cations such as iron, aluminum, and sodium.^1^

Asbestos has been employed in different production sectors of the manufacturing industry due to its physical and chemical properties of heat resistance, even in high temperatures, low density, flexibility, mechanical and chemical resistance, and also because of its low cost.

Inhaled fibers can cause a spectrum of diseases, including lung cancer; malignant mesothelioma of the pleura, peritoneum, pericardium, and tunica vaginalis; laryngeal and ovarian cancers; nonmalignant pleural diseases; asbestosis; and airflow obstruction.^2^ ^,^ ^3^ Less consistent evidence has shown that they are associated with a higher incidence of retroperitoneal fibrosis.^3^ Asbestos exposure is the main risk factor for occupational cancer globally and a significant cause of disease and disability.^4^

The incidence and prevalence of those diseases are intimately related to occupational and environmental exposure to asbestos, such as workers in asbestos mining and processing, as well as workers in industries of manufactured goods such as asbestos-cement, automotive parts, textile products, thermal insulation materials, and others. Relatives of exposed workers and people living in communities surrounding mining and industrial areas also face risk of exposure.^5^ ^,^ ^6^

There are no serological markers nor other types of markers for the early diagnosis of the diseases related to asbestos exposure mentioned above. For pleuropulmonary diseases, the object of the present document, chest imaging is the detection method used globally.

In Brazil, despite efforts to restrict asbestos production and use, the number of reported cases of asbestos-related diseases is still much lower than the estimates,^7^ ^,^ ^8^ which strengthens the need to develop structured programs for the screening of these diseases featuring the incorporation of more sensitive imaging methods such as chest CT.^9^ ^-^ ^13^

## OBJECTIVE

To develop recommendations for the screening of pleuropulmonary diseases related to asbestos exposure through the performance of imaging tests.

## METHODS

A narrative review of the literature of diagnostic imaging of nonmalignant asbestos-related diseases and lung cancer screening in asbestos-exposed individuals was elaborated. The literature used in the development of this document encompassed peer-reviewed publications and documents from national and international institutions. Based on this narrative review, a panel of specialists composed of pulmonologists and a radiologist with expertise in the field proposed recommendations for the diagnosis and monitoring of asbestos-exposed individuals.

## EPIDEMIOLOGY

Asbestos exposure is one of the main occupational risk factors for respiratory diseases and has the greatest impact on morbidity and mortality. Global estimates for 2019 reveal that 239.3 thousand deaths and 4.189 million disability-adjusted life-years derive from asbestos exposure.^4^ The greatest impact is associated with lung cancer, in which asbestos accounts for approximately 10% of global deaths, not to mention thousands of cases of mesothelioma of serous membranes diagnosed on an annual basis.^4^ ^,^ ^14^ Global estimates for the same year suggest that lung cancer has the highest incidence (2.26 million) and the highest mortality (2.04 million) among cancers.^4^ ^,^ ^15^

In Brazil, a cross-sectional study involving former asbestos-cement workers who had been employed predominantly in the 1960s and 1970s found a high prevalence of nonmalignant asbestos-related diseases and a progressive reduction in prevalence among those employed in the 1980s,^16^ a predictable trend due to pressures for asbestos ban, which caused a slow reduction in asbestos use. Data from other countries^17^ and global data^14^ have indicated a slower reduction in mortality, varying between countries and with a greater predicted impact from 2030 onwards. An ecological study suggested greater mortality due to lung cancer in men and women, from mesothelioma in men, and from ovarian cancer in women in a cluster of municipalities that housed asbestos-cement factories and/or asbestos mining in Brazil,^18^ corroborating another study that found evidence of an important underreporting of cases of asbestos-related diseases in the country.^19^

It is important to bear in mind that smoking is the main risk factor for lung cancer, followed by asbestos exposure and air pollution,^4^ ^,^ ^15^ and it is well established that exposure to asbestos and tobacco smoke presents a positive synergism, that is, the associated risk of both exposures is higher than the sum of the respective risks for lung cancer incidence.^20^ ^,^ ^21^ The prevalence of the sum of smokers and former smokers in Brazil and globally exceeds 40% of the population older than 18 years, being higher in the male sex,^22^ ^,^ ^23^ that is, a large number of adult workers may have been exposed to the two risk factors: asbestos and smoking.

Studies indicate that occupational asbestos exposure is associated with a relative risk for lung cancer incidence that is 2 to 10 times higher compared with the general population, and has a dose-response relationship with fiber concentration in the work environment and cumulative exposure,^24^ not to mention the synergistic effect with other carcinogens, such as those present in tobacco smoke.^20^

## IMAGING METHODS

Chest radiography is the exam required by the Brazilian legislation on occupational safety and medicine^25^ for the periodical monitoring of workers exposed to mineral dust. Periodical chest radiographs have the advantages of standardized interpretation through the International Labour Organization’s International Classification of Radiographs of Pneumoconioses criteria^26^ and low radiation dose. Although it is widely used and complies with the Brazilian legislation on occupational safety,^25^ chest radiography has lost relevance with the advent of CT, especially with the drastic reduction in ionizing radiation enabled by the new tomography machines.^27^

It is well established that chest CT is more sensitive for the diagnosis of diseases related to asbestos exposure.^9^ ^,^ ^13^ HRCT, including low-dose CT of the chest,^28^ provides a more accurate diagnosis of interstitial lung diseases, such as asbestosis, and that of pleural thickening, such as pleural plaques. Furthermore, it is more indicated for the early diagnosis of pulmonary nodules. Between 20% and 50% of pleural abnormalities visualized in autopsies and CT scans are not visualized in radiographs, and 15-30% of individuals with radiographs interpreted as normal present abnormalities suggestive of asbestosis on HRCT. In addition to greater sensitivity and specificity, chest HRCT also presents lower variability between experienced chest radiograph readers compared with radiography.^2^ ^,^ ^9^ ^-^ ^13^ ^,^ ^16^ ^,^ ^29^ The patterns of tomographic images also allow to enhance the differential diagnosis between asbestosis and interstitial lung diseases of other etiologies^30^ ^,^ ^31^ and a greater accuracy in the identification of pulmonary nodules.^32^ ^,^ ^33^

In the current scenario, most individuals exposed to asbestos have lower exposure doses because they started working in the 1980s, the decade when movements and actions to restrict and eliminate asbestos use began. Even though such movements and actions had a reduced reach, they succeeded in bringing about improved environmental control and prohibiting the use of amphiboles. As a consequence, exposed individuals may present subtle abnormalities, both in the pleura and in the parenchyma. In addition, individuals with a smoking history, emphysema, and/or chronic bronchitis or other tobacco-related lung diseases, advanced age, heart failure, obesity, and other exposures to dust or particles may present radiographic abnormalities that hinder the adequate identification of asbestosis and reduce the accuracy of interpretation.^2^ ^,^ ^13^ ^,^ ^30^ ^,^ ^31^ ^,^ ^34^ ^,^ ^35^

Specialty societies in the thoracic area have long been indicating the use of chest HRCT to diagnose interstitial lung diseases.^36^ Therefore, there is no justification for indicating HRCT for the diagnosis and monitoring of interstitial diseases in general and restricting it in the case of occupational interstitial diseases like asbestosis.

On the other hand, the identification of nonmalignant abnormalities, especially asbestosis,^21^ but also pleural plaques,^37^ ^,^ ^38^ enables to evaluate the inclusion of these individuals in the high-risk group for developing lung cancer and to ensure that they are monitored.

Furthermore, studies carried out in the past 20 years have shown that the sensitivity of low-dose HRCT (LD-HRCT) to detect interstitial lung abnormalities is apparently similar to that of conventional thin-slice tomography (HRCT), and both of them are superior to the older, initial conventional tomography,^38^ ^-^ ^42^ with lower exposure to radiation. A study involving 2,760 nuclear weapons workers potentially exposed to asbestos found that LD-HRCT enabled the detection of 3.7 times more pleural plaques and five times more interstitial lung diseases than chest radiography.^33^

These results stimulated the conduction of studies focusing on long-term screening for the early diagnosis of lung cancer,^25^ which demands repeated tests with the use of LD-HRCT and, more recently, ultra-LD-HRCT.^(^ ^41^ ^-^ ^44^ In the past, the radiation dose from a conventional chest tomography used to be higher than the dose from 100 chest X-rays. Comparative studies have shown that exposure per exam has been reduced with the use of low-dose helical tomography equipped with a higher number of detectors (32 or more) and new algorithms for image reconstruction. Such studies have found radiation exposures, measured in millisieverts (mSv), of 0.16 mSv and 1-2 mSv from ultra-low-dose HRCT and low-dose HRCT, respectively, compared with 0.05 mSv and 0.24 mSv from lateral and posteroanterior chest X-ray, images being acquired with appropriate quality.^43^ ^-^ ^46^ The risk of HRCT is not zero, but it is much lower than the benefits related to the reduction in mortality by lung cancer revealed by many studies conducted with risk populations. Although it is not possible to produce accurate estimates, estimated risk^47^ is calculated based on the radiation effects of the exposure to the atomic bombs in Hiroshima and Nagasaki. Thus, the estimated risk is that the annual exposure of an individual from the age of 50 to the age of 75 years to LD-HRCT radiation is 1.8% (95% CI: 0.5-5.5%), much lower than the mortality reduction found in different studies, which ranges between 15% and 30%.^27^ ^,^ ^48^ ^-^ ^50^

As studies have shown a favorable inclination towards the utilization of HRCT in lung cancer screening, its use started to be recommended in the USA,^48^ ^-^ ^50^ ^)^ in European countries,^51^ ^)^ by Collegium Ramazzini,^27^ and, recently, by the Brazilian Thoracic Society,^52^ for individuals who meet the criteria suggested in the studies, centered on the main risk factor: smoking. One of the concerns in screening studies is the overdiagnosis of non-neoplastic nodules, leading to procedures that have a negative impact on patients. However, analyses of many international and Brazilian studies have shown only a few relevant complications and that the benefits outweigh the risks.^50^ ^-^ ^52^

The lung cancer risk of individuals with a history of occupational asbestos exposure is comparable to or higher than that of individuals who meet the classic eligibility criteria for lung cancer screening programs with LD-HRCT^49^ ^,^ ^53^ ^,^ ^54^ even if they have been former smokers for more than 15 years or have smoked less than 20 pack-years. A recent guideline from the American Cancer Society^49^ ^,^ ^50^ recommends, among other updates, that the number of years since smoking cessation should not be one more eligibility criterion for inclusion in screening programs, which is something that other studies, such as those that suggest the use of risk calculators,^55^ ^-^ ^57^ already do.

A model developed in England to assess risk prediction based on a cohort study involving more than 18 million individuals revealed that asbestos exposure is one of the main risk prediction factors for lung cancer.^57^

## RECOMMENDATIONS

1. For all individuals with a history of occupational exposure to asbestos for at least one year, or domestic exposure (workers’ relatives exposed through clothes that are likely to be contaminated or exposed to asbestos products brought for use inside the domicile) or environmental exposure (individuals living near mining companies or factories involved in the manufacture of asbestos products) for at least five years, with 20 years’ latency or over, the recommendation is that, apart from clinical, functional assessment, and the necessary compliance with the labour rules for periodical examination, when needed, they should be submitted to an HRCT of the chest as the first imaging test (Table 1).

**Table 1:** Summary of recommendations.

Eligibility criteria	Recommendations
Occupational exposure to asbestos ≥ 1 year, or domestic/environmental exposure to asbestos ≥ 5 years AND Latency period ≥ 20 years	Submit to HRCT as the first imaging test
Age 50-75 years old AND Exposure to asbestos AND Any nonmalignant asbestos-related disease: Pleural plaques, diffuse pleural thickening, round atelectasis, and/or asbestosis	Submit to pulmonary function test with plethysmography and DL_CO_ assessment, if available
Age 50-75 years old AND Exposure to asbestos with or without nonmalignant asbestos-related disease AND Latency period ≥ 20 years AND Lung cancer risk estimate > 1.5%*	Submit to annual lung cancer screening with LD-HRCT Lung-RADS diagnostic criteria are recommended as the main method of assessment of LD-HRCT findings Lung cancer screening programs should have support of smoking cessation programs

LD: low dose; Lung-RADS: Lung Imaging Reporting and Data System.^61^ ^)^ *Estimated by the Liverpool Lung Project (LLP)^55^ ^,^ ^56^ or the CanPredict^57^ cancer risk calculators.

Based on the findings obtained through the images and on clinical and functional aspects, the individuals can be followed up through criteria defined according to the potential risk, as follows:

1.1. Individuals aged 50 or older up to 75 years of age presenting pleural plaques and/or diffuse pleural thickening, with or without round atelectasis, and/or signs compatible with asbestosis, mainly presence of subpleural dots and lines, interlobular septal thickening, parenchymal bands, ground-glass opacities, mosaic perfusion in early cases, and in more advanced cases, also traction bronchiectasis and honeycombing,^2^ ^,^ ^58^ ^,^ ^59^ in addition to a previous clinical assessment, should be submitted to an assessment of the respiratory function, which should be thorough whenever possible (not only spirometry), with determination of DL_CO_. If they meet the inclusion criteria, such as absence of comorbidities that impose limitations on diagnosis and treatment procedures in the case of a cancer diagnosis, they should be followed up according to item 1.2 below.

1.2. Annual lung cancer screening is recommended to the services that meet the requirements suggested in the studies and recommendations.^27^ ^,^ ^49^ ^-^ ^52^ ^,^ ^60^ Such services should plan the timely performance of tests, including reassessments in accordance with the indication of the nodules found, and the performance of procedures for diagnosis, follow-up, and treatment with the use of LD-HRCT to minimize the risks of radiation exposure.

2. Individuals aged 50 or older up to 75 years of age with occupational exposure to asbestos for one year or over or domestic and/or environmental exposure for five years or over, with 20 years’ latency or over, for any of the exposure conditions, even if they do not present asbestos-related diseases at the moment, should be considered exposed in a significant way and assessed through the use of risk calculators for inclusion in the screening program with the use of LD-HRCT of the chest. If they do not meet such criteria, they can be included in the screening program through the other factors assessed by the calculators.

3. Chest LD-HRCT screening should be performed in individuals with a history of occupational exposure to asbestos who meet the criteria described above if their lung cancer risk estimate is at least 1.5% according to Liverpool Lung Project (https://liverpoollungproject.org.uk/MLRV3/MLRCalculation.html) or CanPredict calculators.^55^ ^-^ ^57^ ^1^

4. For the assessment of CT scans in the lung cancer screening program, the classification of findings and diagnosis criteria recommended by Lung Imaging Reporting and Data System (Lung-RADS),^61^ a tool recommended in lung cancer screening programs,^51^ ^,^ ^54^ ^-^ ^57^ should be used.

5. All the services involved in lung cancer screening programs should have the support of smoking cessation programs.

## FINAL CONSIDERATIONS

The diagnosis and registration of occupational diseases have historically been inadequate and limited for many reasons, such as the deficient education of health professionals and the lack of specialized services in Brazil. Respiratory diseases deriving from asbestos exposure are included in this context. Diagnosing asbestos-related diseases is necessary to enhance the monitoring of patients’ health. Furthermore, with such a diagnosis, Brazil can have accurate knowledge of the repercussions of asbestos use, and the victims will have the right to seek compensation if they wish to, either from the State (social security, environmental care) or from the companies that generated the exposure.

The present document represents the position of the Committee on Environmental and Occupational Diseases of the Brazilian Thoracic Society on screening, diagnosis, and follow-up of asbestos-related diseases with the main objective of improving their recognition and monitoring.

## Footnotes

1 The risk calculators Liverpool Lung Project (LLP)^54^ ^,^ ^55^ and CanPredict^56^ are tools developed to estimate individual risk of lung cancer in a time horizon of five to ten years. They include asbestos exposure and smoking in their mathematical models, as well as clinical and demographic variables.
